# ^1^H Nuclear Magnetic Resonance: A Future Approach to the Metabolic Profiling of Psychedelics in Human Biofluids?

**DOI:** 10.3389/fpsyt.2021.742856

**Published:** 2021-12-13

**Authors:** Sylvana Vilca-Melendez, Malin V. Uthaug, Julian L. Griffin

**Affiliations:** ^1^Department of Brain Sciences, Faculty of Medicine, Imperial College London, London, United Kingdom; ^2^The Centre for Psychedelic Research, Department of Brain Sciences, Faculty of Medicine, Imperial College London, London, United Kingdom; ^3^Department of Neuropsychology and Psychopharmacology, Faculty of Psychology and Neuroscience, Maastricht University, Maastricht, Netherlands; ^4^Department of Metabolism, Digestion and Reproduction, Faculty of Medicine, Imperial College London, London, United Kingdom

**Keywords:** psychedelics, metabolomics, proton nuclear magnetic resonance, mass spectrometry, liquid chromatography, psilocybin, LSD, new psychoactive substances

## Abstract

While psychedelics may have therapeutic potential for treating mental health disorders such as depression, further research is needed to better understand their biological effects and mechanisms of action when considering the development of future novel therapy approaches. Psychedelic research could potentially benefit from the integration of metabonomics by proton nuclear magnetic resonance (^1^H NMR) spectroscopy which is an analytical chemistry-based approach that can measure the breakdown of drugs into their metabolites and their metabolic consequences from various biofluids. We have performed a systematic review with the primary aim of exploring published literature where ^1^H NMR analysed psychedelic substances including psilocin, lysergic acid diethylamide (LSD), LSD derivatives, *N,N*-dimethyltryptamine (DMT), 5-methoxy-*N,N*-dimethyltryptamine (5-MeO-DMT) and bufotenin. The second aim was to assess the benefits and limitations of ^1^H NMR spectroscopy-based metabolomics as a tool in psychedelic research and the final aim was to explore potential future directions. We found that the most current use of ^1^H NMR in psychedelic research has been for the structural elucidation and analytical characterisation of psychedelic molecules and that no papers used ^1^H NMR in the metabolic profiling of biofluids, thus exposing a current research gap and the underuse of ^1^H NMR. The efficacy of ^1^H NMR spectroscopy was also compared to mass spectrometry, where both metabonomics techniques have previously shown to be appropriate for biofluid analysis in other applications. Additionally, potential future directions for psychedelic research were identified as real-time NMR, *in vivo* ^1^H nuclear magnetic resonance spectroscopy (MRS) and ^1^H NMR studies of the gut microbiome. Further psychedelic studies need to be conducted that incorporate the use of ^1^H NMR spectroscopy in the analysis of metabolites both in the peripheral biofluids and *in vivo* to determine whether it will be an effective future approach for clinical and naturalistic research.

## Introduction

While psychedelics have demonstrated a possible therapeutic potential for treating mental health disorders such as depression (1–5) and treating autoimmune disorders (6–8), further research is needed to understand their biological effects and mechanisms when considering the development of future novel therapeutic approaches. Some results observed in previous psychedelic studies could be attributed to other non-pharmacological factors (i.e., set and setting and/or therapeutic relationship between patient and therapist) (9–14). Thus, biological evidence is needed to corroborate the effect of psychedelics on various mental health related parameters. This is where a more chemistry-based/-omic approach is favourable as the introduction of a psychedelic substance into the human system is fundamentally a *chemical interaction* between these molecules and their respective receptors in the body. Factors such as the psychedelic molecule concentration and the receptor binding efficacy could potentially result in a difference in physiological and psychological experience (15). Another important factor is the effect that metabolism can have in chemically modifying and converting psychedelic molecules into different resulting molecules (16). All these factors need to be monitored to understand the full extent of the effect psychedelics have on human health.

The monitoring of drug metabolism is primarily an aspect of pharmacology; however, metabolomics could help understand the pharmacology significantly in terms of the pharmacokinetics (i.e., how quickly the drug is absorbed and metabolised) and its metabolic consequences (i.e., what metabolites have been produced from this metabolism). The implications of the collection of data related to drug metabolism could potentially aid in increased understanding of an individual's subjective experience with a psychedelic and their predisposition to adverse effects (and so too how to reduce such adverse effects). Furthermore, it could bring about the emergence of a novel and objective method rooted in biology to monitoring treatment progress and response over time and that if used together with subjective reports and neuroimaging could be a more holistic approach to measure the effects of psychedelics and the mechanism in which they exert their effects.

### NMR Spectroscopy

One of the tools with major potential to investigate the biological effects and pharmacodynamics of psychedelics is the metabonomics application of proton nuclear magnetic resonance (^1^H NMR) spectroscopy. NMR spectroscopy is a powerful technological tool capable of determining the structure of a molecule (17). It is one of the most important spectroscopic tools in chemistry, having been the source of multiple Nobel Prizes in the last century (18). It was in 1945, that two independent physics groups, Bloch, Hansen & Packard at Stanford University and Purcell, Torrey and Pound at Harvard University, were successful in observing the phenomenon of nuclear magnetic resonance in solids and liquids (19). Its importance increased and in 1991, the Swiss physical chemist Richard R. Ernst was awarded the Nobel Prize in Chemistry for his outstanding contribution to the development of the experimental NMR technique for structure determination of biological macromolecules (19).

NMR spectroscopy relies on certain isotopes of nuclei that possess the quantum mechanical property of spin which causes the nuclei to adopt different energy states when placed in a magnetic field (19). Nuclei that have a spin of zero do not exhibit the NMR effect but nuclei that have spin values of ½ or greater can adopt different energy levels in the magnetic field which can be detected when a radiofrequency is applied to perturb the system (19). The most common isotopes observed by NMR are hydrogen-1 isotope (^1^H), which is often referred to as a proton, and carbon-13 (^13^C) (20). There are also other isotopes that can be used; including nitrogen-15 and phosphorous-31 (19), but their application is relatively rarer in NMR spectroscopy, in part reflecting their lower abundance in many organic compounds and smaller gyromagnetic ratio (a property that in part determines the magnitude of the NMR signal) (20). All organic molecular structures have a hydrocarbon skeleton that forms the “backbone” of the molecule; it is the substitution with other elements alongside the rearrangement of these hydrogen and carbon atoms that produce unique structures of the organic molecules. It is this abundance of hydrogen and carbon that drives the use of ^1^H NMR and ^13^C NMR over the other NMR-active isotopes in biochemistry.

NMR spectroscopy analysis can detect the unique environment of the NMR-active atoms in the molecule to produce an NMR spectrum. Each peak that is detected in the NMR spectrum has an area under the curve that relates to the concentration of the molecule, while the exact frequency (referred to as the chemical shift) contains structural information (19). In an NMR spectrum, the X-axis corresponds to the frequency axis and provides the chemical shifts in ppm (which allows the comparison of relative frequency changes at different magnetic field strength), and the Y-axis is the signal intensity (20). This simple spectrum is referred to as one-dimensional (1D) NMR spectrum. It is through the interpretation of the spectrum that the molecular structures in the sample being analysed can be predicted. In addition to chemical shift, the frequency of a given nuclei is also perturbed by adjacent nuclei, creating a splitting pattern, referred to as J-coupling, which holds further structural information (19).

Over time there has been additional improvements made to data analysis processing and one such advance was the introduction of two-dimensional (2D) NMR spectroscopy. In a 2D NMR spectrum there are two frequency axes, rather than the single frequency axis. The major advantage of 2D NMR over 1D NMR analysis is that it has the ability to distinguish between overlapping signals, reducing spectral crowding (21) which is often a problem in the NMR analysis of larger molecules. 2D NMR is particularly useful in structural elucidation, especially when trying to determine the three-dimensional structure of proteins. There are specific types of 2D NMR spectroscopy (20, 22, 23), including correlation spectroscopy (COSY), total correlation spectroscopy (TOCSY), nuclear Overhauser effect spectroscopy (NOESY), rotating frame Overhauser effect spectroscopy (ROESEY), heteronuclear single quantum coherence (HSQC) spectroscopy and heteronuclear multiple bond correlation (HMBC) spectroscopy. Each type of 2D NMR produces unique data and is employed dependent on the spectral information required for the experiment design.

### The Application of NMR to Metabonomics

Metabolism describes the chemical transformations of food components and other exogenous substances that are carried out by an organism to maintain life. The molecules that are produced from these subsequent chemical reactions are called metabolites (16) and are necessary for the function of all living tissue and cells. The field of metabolomics holistically investigates these metabolic factors to observe the effect it has on health and biological response in an organism; this is achieved by detecting, identifying and accurately measuring the concentration of metabolites (24). Metabonomics describes a subset of metabolomics and though both approaches are similar in their use of analytical techniques; the metabonomic approach places greater emphasis on measuring the changes in metabolite across a specified measurement of time and in response to a pathophysiological change or genetic modification (25). The most commonly used methods for metabolically profiling the impact of drugs are through the use of spectroscopic tools like mass spectrometry (MS), NMR spectroscopy and chromatographic methods like high performance liquid chromatography (24, 26, 27). These tools allow for the accurate high-throughput screening of hundreds to thousands of metabolites and have been most often applied to biofluids, including blood plasma, urine, and saliva (24, 28). It can provide metabolic information from individuals or populations, which can then be applicable in personalised medical treatment or public-wide healthcare interventions (27).

Aside from metabolic profiling, NMR can also be used in other scientific research areas. It is important in structural biology for drug discovery (29–33) and has been described as the “gold standard” tool (22) for structure determination. One example of NMR spectroscopy has been in fragment-based drug design (FBDD) (34–36). This combines molecular fragments that are selected from a fragment library (37) of molecules that are known to bind to specific targets, producing a potential novel drug. NMR spectroscopy is required in the design and screening of the FBDD library and to analyse the fragment purity (38). So far NMR has successfully identified novel molecules in multiple FBDD drug discovery experiments (39–44). NMR can also be used in chemistry for the structural elucidation of a molecule (45–47). NMR can even be used in quality assessment for the food industry (48, 49) and petroleum industry (50, 51). Occasionally some of these purposes will overlap, for example natural products chemistry involves both drug discovery and structural elucidation as it identifies novel molecules for pharmaceutical treatment that are derived from natural products (i.e., plants and living organisms) using NMR (52). By considering all the different applications of NMR, this review will determine the most used application of NMR in psychedelic research to date.

### Psychedelic Research

Psychedelic molecules can be found in abundance in nature, such as in for example psilocybin mushrooms, the ayahuasca vine named *Banisteriopsis caapi* or the mescaline containing cacti known as peyote (53). Some of these natural substances have been used for thousands of years in ceremonies and religious rituals across cultures in different locations in the world (53). In fact, the study of natural products chemistry (*i.e*., the study of molecules produced from living organisms like animals, fungi and plants) have given rise to psychedelic psychoactive molecules being isolated and then chemically synthesised in the laboratory (52, 54, 55). Additionally, once the molecular structure of a psychedelic molecule had been established, synthetic chemistry could be used to alter the functional groups on these molecules producing novel psychedelic derivatives (56–58). A notable example was when the Swiss chemist Albert Hofmann synthesised lysergic acid diethylamide (LSD) for the first time in 1938 (59); this molecule is a derivative of lysergic acid found in ergot fungus.

**Figure 1:**
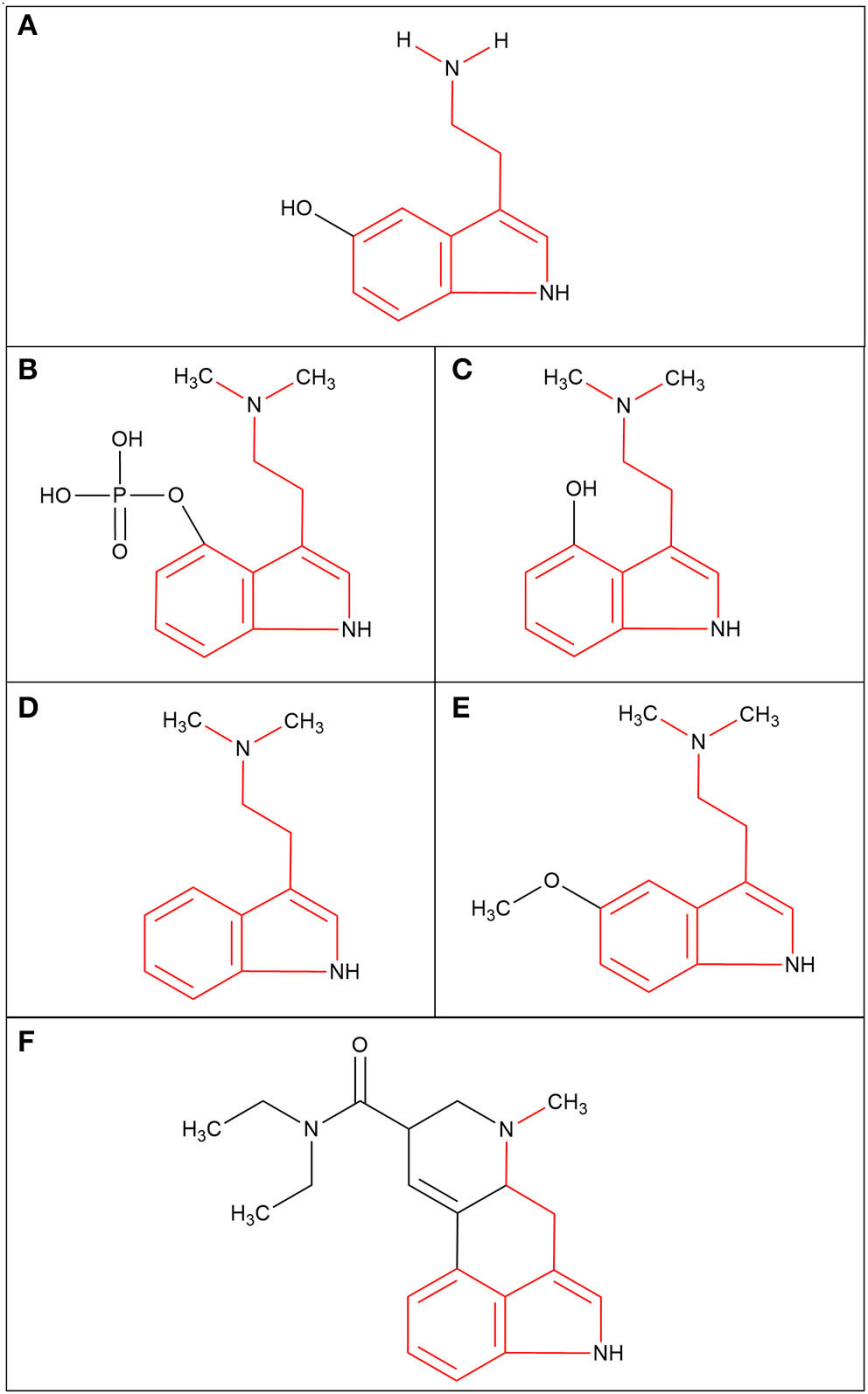
The molecular structures of the serotonin neurotransmitter and the indolealkylamines class of psychedelic molecules (tryptamine moiety highlighted in red). **(A)** Serotonin; 5-hydroxytryptamine (5-HT). **(B)** Psilocybin; 4-phosphoryloxy-N,N-dimethyltryptamine (4-PO-DMT). **(C)** Psilocin; 4-hydroxy-N,N-dimethyltryptamine (4-OH-DMT). **(D)** N,N-dimethyltryptamine (DMT). **(E)** 5-methoxy-N,N-dimethyltryptamine (5-MeO-DMT). **(F)** Lysergic acid diethylamide (LSD).

Psychedelic molecules can be separated into two major groups: indolealkylamines and phenylethylamines (60). All indolealkylamines share the basic structure of a tryptamine moiety and are often referred to as belonging to the tryptamine class (60). The tryptamine moiety is also the structural backbone present in serotonin (5-hydroxytryptamine, 5-HT) (53), as seen in Figure 1A. 5-HT is an essential neurotransmitter that has been implicated in mood regulation, learning mechanisms and decision making (61, 62). This moiety is also present in psilocin, psilocybin, N-N-Dimethyltryptamine (DMT), 5-methoxy-N-N-Dimethyltryptamine (5-MeO-DMT) and LSD, as seen in Figure 1. Despite psilocybin being the main compound in *psilocybe* mushrooms, it is often falsely assumed to be the active molecule that binds to the receptors. Psilocybin has to be dephosphorylated by phosphatase to become psilocin, and it is psilocin that is the active metabolite that produces the psychoactive effect in the brain (60). Therefore, both structures are included for comparison (Figures 1B,C) and both will be included in this review. It should be noted that the indolealkylamines group can be divided further into two subgroups called simple tryptamines and ergolines (63), and this provides more clarity about the structure. Simple tryptamines like psilocin, DMT and 5-MeO-DMT have conformational flexibility (60), this is where a molecule can adjust its molecular shape as it interacts with environmental factors or receptors. Contrastingly, ergolines like LSD have relatively rigid structures (53) because of the presence of a number of conjugated double bonds across the ring structures (Figure 1F), which means that the LSD molecule cannot rotate freely around bonds (i.e., freely adjust its shape). It is important to note that there are hundreds of LSD derivatives that have been chemically synthesised since the discovery of LSD (64); these are often referred to as new psychoactive substance (NPS). These derivatives often become available on the illegal drug market as “designer drugs” and are consumed with the assumption they will evoke a similar biological response as LSD. Phenylethylamine psychedelics are often considered NPSs too (65), but their molecular structures are vastly different from traditional indolealkylamine psychedelics. This is because phenylethylamines are derived from the molecule mescaline found in the peyote cactus (66). Unfortunately research into this group of psychedelics is less common compared to indolealkylamines.

It is understood that psychedelics are agonists or partial agonists for the brain serotonergic 5-HT_2A_ receptors, which are expressed on the apical dendrites of the neocortical pyramidal cells in layer V (53, 67). The tryptamine moiety is an essential part of psychedelic molecules because it is the part that binds to the orthosteric pocket of the 5-HT_2A_ receptor (68), which is the endogenous agonist binding site. The 5-HT_2A_ receptors are believed to play a critical role in higher cortical brain functions (69). However, there is also evidence that there is serotonergic activity outside the central nervous system (CNS). Approximately 90% of the body's 5-HT is produced by enteroendocrine cells that reside in the epithelium of the gut (70). 5-HT is fundamental for the formation and maturation of the Enteric Nervous System (ENS) and has recently demonstrated that the gut microbiota plays an important role in the modulation and the function of the ENS (70). Additionally, a recent paper implicates the gut microbiota in the modulation 5-HT_2A_ receptors that promote activity in the brain (71), and another paper found that mice that were given a combination of antibiotics experienced cognitive impairments in recognition tasks (72). If psychedelic molecules are found to interact with the gut microbiome this could lead to a modulation of the ENS and CNS, therefore this interaction needs to be considered when investigating the physiological and psychological effect of psychedelic molecules.

### The Application of Psychedelics in Disease Treatment

Recent research into psychedelics as a therapeutic approach appears to be promising. Previous clinical research have shown that oral doses of psilocybin may improve mental health related variables and produce persistent positive psychological effects for depression and anxiety (1–5). Psychedelics may be most beneficial when combined with psychotherapy programmes which has led to the manifestation of the approach known as *psychedelic-assisted psychotherapy* (73, 74). The results of all these studies may have important implications for patients with for example treatment-resistant depression (TRD) that are not experiencing any amelioration in their symptoms despite going through cognitive behavioural therapy or anti-depressant drug therapy (74–79). Importantly, there is a strong association between depression and suicidal ideation (80–82). In fact, 30% of TRD patients attempt suicide at least once during their lifetime (80). Thus, novel treatment options are highly warranted. For TRD patients, psychedelic assisted psychotherapy may be such an option. However, this warrants further research.

Interestingly, previous research suggests that psychedelics could potentially ameliorate autoimmune diseases (AiD) (6). Through activating NF-κB and mitogen-activated protein kinases, the psychedelic molecules are believed to have a downstream effect on the resulting signalling pathways involved in the inflammation, cell survival and cell proliferation (8). Research has even implicated DMT and 5-MeO-DMT in the modulation of the sigma-1 receptor found on human primary monocyte-derived dendritic cells (7). It has been speculated that because these cells are involved in the inflammatory and infection response, the psychedelic molecules play a role in their regulation of immune homeostasis (7). Even though, future studies need to be conducted to fully understand the immunomodulatory mechanisms of psychedelics, the current scientific knowledge reservoir suggests it has potential as a future treatment for AiD (6). Moreover, because there are a multitude of diseases that also involve inflammation dysfunction, there is even the potential for psychedelic studies to focus on potential treatment of other autoimmune related disorders such as arthritis, atherosclerosis and Alzheimer's disease (8).

The recent wave of research taking place on psychedelic substances may be a testament to the shift in opinion that this class of molecules could be beneficial, rather than harmful, for physical and mental health treatment (83). Nevertheless, more research is warranted before the adoption of these novel interventions. Ultimately, it is beneficial to assess the integration between the field of metabonomics and the field of psychedelic research to ensure appropriate advancements are being made in the field. This is to the best of our knowledge the first review paper on ^1^H NMR spectroscopy use in psychedelic research. Thus, the first aim of this review is to explore the use of ^1^H NMR spectroscopy in psychedelic research. This was done so to determine whether ^1^H NMR spectroscopy has ever been used to study the human metabolic response to psychedelic substances with biofluid analysis. The second aim of this review was to assess the potential benefits and limitations associated with ^1^H NMR spectroscopy in this research field. Finally, the third aim of this review will be to suggest novel applications of ^1^H NMR that may improve the analytical data retrieved from future psychedelic research studies.

## Methods

We collected publications from the PUBMED database and Cochrane Library database. The search was conducted March-May 2021. These databases were used in combination to acquire a wider collection of publications, but no further databases were used to reduce duplicates during screening. The following terms were searched: *proton nuclear magnetic resonance* or *1H NMR*, and *psychedelics, psilocybin, lysergic acid diethylamide, dimethyltryptamine, 5-methoxy-N,N-dimethyltryptamine*. Any publications that included LSD derivatives were added. There were no publications that included phenylethylamines found through this search. No limits were set regarding the publication date. The search identified a total of 87 papers (84 from PUBMED and 3 from Cochrane). Subsequent examination of reference lists identified other eligible studies for this review. Fourteen papers were acquired through this method. This brought the total amount of papers to 102. Papers were excluded if they were not written in English, investigated non-psychedelic molecules (e.g., cannabis or opioids) or were conducted using irrelevant methods (e.g., functional magnetic resonance imaging, *etc*.). The screening of publication titles and abstracts led to 79 papers being excluded, leaving 23 that were screened in more detail for eligibility. These final 23 papers were used in this review and are listed in Table 1.

**Table 1:** Summary of all the publications included in this review.

**References**	**Molecules analysed**	**Spectroscopic/Chromatography tools**	**The use of NMR in study**
Sherwood et al. (84)	5-MeO-DMT	^1^H NMR, HPLC	Analytical profiling of 5-MeO-DMT in a freebase salt form
Brandt et al. (85)	ECPLA (LSD isomer)	^1^H NMR, *^13^C NMR*, MS, LC, GC, GC-sIR	Analytical profiling for ECPLA molecule so that it can be detected in future research
Cozzi and Daley (86)	DMT hemifumarate (DMT salt base)	^1^H NMR, *^13^C NMR, GC-MS, HPLC*	Synthesis and analytical profiling of DMT in a hemifumarate salt form, this form to be used for intravenous administration
Amariz et al. (87)	DMT	^1^H NMR, GC-MS	Identification and quantification of DMT present in the *Mimosa tenuiflora* bark
Brandt et al. (88)	1CP-LSD (LSD isomer)	^1^H NMR, *^13^C NMR*, MS, LC, GC, GC-MS, GC-IR	Analytical profiling for 1CP-LSD as this novel isomer may have psychoactive effects in future clinical trials investigation
Tsochatzis et al. (89)	1B-LSD (LSD isomer)	^1^H NMR, *^13^C NMR*, GC-MS, LC-MS	Identification of 1B-LSD from an unknown substance on blotting paper that was seized
Lenz et al. (90)	PsiP, PsiL (psilocybe enzymes)	^1^H NMR, *^13^C NMR, MS, IR*	Monitoring enzymes from the *psilocybe cubensis* mushroom as they react with psilocybin molecule to produce psilocin
Blei et al. (91)	Harmane, Harmine, β-carbolines (psilocybe species)	^1^H NMR, *^13^C NMR*, LC-MS, HPLC	Analytical profiling of the secondary metabolome psilocybe species that were present in the psilocybe mushroom
Brandt et al. (92)	1B-LSD (LSD isomer)	^1^H NMR, *^13^C NMR*, MS, LC, GC, GC-IR	Analytical profiling for 1B-LSD as this novel isomer may have psychoactive effects in future clinical trials investigation
Pereira et al. (93)	DMT	^1^H NMR, *^13^C NMR, MS*	Identification and quantification of DMT present in the *Erythroxylum pungens* bark and leaf extract
Brandt et al. (94)	LSM-775 (LSD isomer)	^1^H NMR, *^13^C NMR*, MS, GC-MS, GC-IR, HPLC	Analytical profiling for LSM-775 as this novel isomer may have psychoactive effects in future clinical trials investigation
Brandt et al. (95)	ETH-LAD, 1P-ETH-LAD (LSD isomers)	^1^H NMR, *^13^C NMR*, MS, GC-MS, GC-IR, HPLC	Analytical profiling for ETH-LAD and 1P-ETH LAD as these novel isomers may have psychoactive effects in future clinical trials investigation
Soares et al. (96)	DMT	^1^H NMR, *^13^C NMR, MS, LC-MS, IR*	Identification and quantification of DMT present in the *Psychotria viridis* leaf extract
Brandt et al. (97)	AL-LAD, LSZ (LSD isomoers)	^1^H NMR, *^13^C NMR*, MS, GC-MS, GC-IR, HPLC	Analytical profiling for AL-LAD and LSZ as these novel isomers may have psychoactive effects in future clinical trials investigation
Brandt et al. (98)	1P-LSD (LSD isomer)	^1^H NMR, *^13^C NMR*, MS, GC, LC, IR	Analytical profiling for 1P-LSD as this novel isomer may have psychoactive effects in future clinical trials investigation
Zhi et al. (99)	Bufotenin, Psilocin, DMT	^1^H NMR, *^13^C NMR*, MS, IR	Identification and quantification of bufotenine, psilocin, DMT present in the *Desmodium elgans* leaf extract
Shoda et al. (100)	PCG (psilocybe metabolite)	^1^H NMR, *^13^C NMR*, MS, HPLC	Analytical characterisation of PCG, a metabolite that is excreted in the urine of psilocybe mushrooms users
Alias et al. (101)	DMT	^1^H NMR, *^13^C NMR, MS, IR*	Identification and quantification of DMT present in the *Fissistigma latifolium* bark
Buchanan et al. (102)	DMT	^1^H NMR, *^13^C NMR*	Identification and quantification of DMT present in the *Acacia confusa* leaf extract
Costa et al. (103)	DMT, Bufotenin	^1^H NMR, *^13^C NMR*, MS, HPLC, IR	Identification and quantification of bufotenin present in the skin secretions of three arboreal amphibian species
Shirota et al. (104)	Psilocin, Psilocybin	^1^H NMR, *^13^C NMR*, ^31^P NMR, IR	Identification of psilocin, psilocybin and additional precursor molecules for the purpose of producing large-scale synthesis
Salamone et al. (105)	LSD, iso-LSD (LSD isomer)	^1^H NMR	Integration of C-9 resonance in LSD and iso-LSD to allow epimerisation study to be conducted
Migliaccio et al. (106)	Bufotenin, Psilocin	^1^H NMR	Analytical profiling for bufotenine and psilocin to allow for the comparison study of molecular properties

*This is organised in order of publication date, from newest to oldest. 5-MeO-DMT, 5-methoxy-N-N-Dimetyltryptamine; ^1^H NMR, proton nuclear magnetic resonance; HPLC, high performance liquid chromatography; ECPLA, N-ethyl-N-cyclopropyl lysergamide; LSD, lysergic acid diethylamide; MS, mass spectroscopy; LC, liquid chromatography; GC, gas chromatography; GC-sIR, gas chromatography-condensed-phase infrared spectroscopy; DMT, dimetyltryptamine; ^13^C NMR, carbon-13 nuclear magnetic resonance; 1CP-LSD, 1-cyclopropanoyl-d-lysergic acid diethylamide; GC-IR, gas chromatography-infrared spectroscopy; 1B-LSD, 1-butanoyl-d-lysergic acid diethylamide; HRMS, high resolution mass spectroscopy; PsiP, psilocybe cubensis phosphatase; PsiL, psilocybe cubensis laccatase; LSD-775, lysergic acid morpholide; ETH-LAD, N^6^-ethyl-6-norlysergic acid diethylamide; 1P-ETH-LAD, 1-propionyl-N^6^-ethyl-6-norlysergic acid diethylamide; AL-LAD, N^6^-alyll-6-norlysergic acid diethylamide; LSZ, (2'S,4'S)-lysergic acid 2,4-dimethylazetidide; 1P LSD, 1-propionyl-d-lysergic acid diethylamide; PCG, psilocin glucuronide; ^31^P NMR, phosphorous-31 nuclear magnetic resonance*.

### Description of Studies

Two research groups used ^1^H NMR spectroscopy during the process of synthesising psychedelic molecules into their water-soluble salt forms. Sherwood et al. (84), synthesised 5-MeO-DMT succinate salt and Cozzi and Daley (86) synthesised DMT hemifumarate salt. The reactions used to synthesis these molecules were different; 5-MeO-DMT succinate salt was produced using a Fischer indole reaction, whereas the DMT hemifumarate salt was produced with a Speeter and Anthony approach. Both studies selected and optimised their synthetic routes to produce the highest yield and highest purity salts. Purity refers to the percentage of active pharmaceutical ingredient (5-MeO-DMT and DMT) present in the salt produced, both salts had a purity above 99.8%. Analytical characterisation of these compounds was achieved using ^1^H NMR spectroscopy, X-ray powder diffraction, thermogravimetric analysis, differential scanning calorimetry. Sherwood et al. additionally used dynamic vapour sorption (DVS) and optical microscopy, while Cozzi and Daley used ^13^C NMR spectroscopy, GC-MS, HPLC, residual solvent analysis by GC headspace sampling, and residual lithium analysis by inductively coupled plasma-MS. The implications of this research were that it could potentially improve the application of high-purity water-soluble salt forms in future clinical trial, 5-MeO-DMT succinate salt used for intramuscular (IM) injection or DMT for intravenous (IV) injection. ^1^H NMR spectroscopy was essential in determining drug purity; however, it was not used as a metabonomics tool.

**Figure 2:**
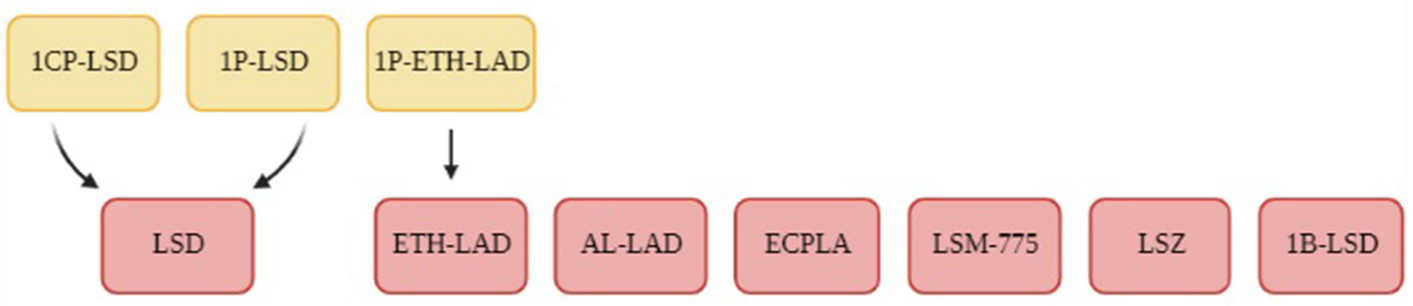
A flowchart of the LSD derivatives that were analyses using ^1^H NMR spectroscopy in the studies conducted by Brandt et al. (85, 88, 92, 94, 95, 97, 98). The LSD derivatives are demonstrated in the red boxes and the yellow boxes highlight potential prodrugs.

Brandt *et al*. conducted their first LSD derivative experiment on 1-Propionyl-d-lysergic acid diethylamide (1P-LSD) (98) in 2016. LSD analogues typically have limited analytical data and so Brandt et al. used ^1^H NMR, ^13^C NMR spectroscopy, various chromatographic and MS methods and IR spectroscopy for their analytically characterisation and comparison to LSD. Building on this first research paper, Brandt et al. published several follow-up papers on additional LSD derivatives using similar analytical tools. The papers included the molecules N6 -allyl-6-norlysergic acid diethylamide (AL-LAD) (97), (2'S,4'S)-lysergic acid 2,4-dimethylazetidide (LSZ) (97), N6 -ethyl-6-norlysergic acid diethylamide (ETH-LAD) (95), 1-propionyl N6 -ethyl-6-norlysergic acid diethylamide (1P-ETH-LAD) (95), lysergic acid morpholide (LSM-775) (94), 1-butanoyl-d-lysergic acid diethylamide (1B-LSD) (92), 1-cyclopropanoyl-LSD (1CP-LSD) (88) and the N-ethyl-N-cyclopropyl lysergamide (ECPLA) (85), as seen in Figure 2. Each molecule mentioned was successfully characterised with the use of ^1^H NMR to understand the molecular structure and chemical properties. Some papers (88, 95, 98) included the incubation of the LSD analogue with human serum for 24 h to observe any molecular changes in this environment. They found that in serum 1P-LSD and 1CP-LSD formed LSD and that 1P-ETH-LAD formed ETH-LAD. This suggests that some LSD derivatives may potentially serve as pro-drugs. Brandt *et al*. also wanted to assess the behavioural characterisation of LSD analogues by injecting the substances into male C57BL/6 J mice and measuring their head twitch response (HTR) for 30 min using magnetometer coil recordings. The control group of mice received saline injections instead. LSD is known to induce HTR which is associated with 5-HT_2A_ receptor activation, and so HTR is an indicator of an LSD-like behavioural effect. Results demonstrated that 1P-LSD, produced a dose-dependent increase in HTR counts and that its potency was significantly less than LSD potency. Additionally, when pre-treatment of the selective 5-HT_2A_ receptor antagonist M100907 was used before 1P-LSD administration there was no HTR. This indicates that 1P-LSD produces LSD-like effect in mice mediated by 5-HT_2A_ receptor activation. AL-LAD and LSZ also produced dose-dependent increase of HTR counts where LSZ was equipotent to LSD in mice and AL-LAD was slightly less potent. Additionally, 1B-LSD and 1-CP-LSD were also found to produce an increase in HTR counts. The only molecule that did not induce HTR was LSM-775. It was only through the pre-treatment of antagonist WAY-100,635 that HTR was present in the mice that were administered LSM-775. This antagonist is known to block 5-HT_1A_ receptors and so it suggests that the LSM-775 activation of 5-HT_1A_ receptors is interfering with HTR. This aligns with the reports that LSM-775 produces a weak LSD-like effects in humans. Brandt *et al*. did not include any metabonomics approach to analyse biofluids in their research overall and so it is clear that NMR spectroscopy was only used to produce an analytical profile for the LSD analogues.

^1^H NMR spectroscopy was used in a study by Buchanan et al. (102) that investigated the Chinese shrub *Acacia confusa*. Similarly, Alias et al. (101) investigated the bark of the *Fissistigma latifolium* plant. Once the molecules were isolated, they underwent structural elucidation using ^1^H NMR accompanied with additional spectroscopic tools like ^13^C NMR, MS and infrared (IR) spectroscopy. Both of the plant materials contained multiple molecules but interestingly in both of these plants the only molecule that was identified of relevance to this review was DMT. Zhi et al. (99) also studied the leaves of the *Desomodium elegans* using the same spectroscopic tools. It found psilocin, DMT and bufotenin to be present, alongside an entirely novel molecule, named desmodeleganine. This discovery was relevant to psychedelic research because this study discovered that desmodeleganine is a monoamine oxidase inhibitor (MAOI). MAOIs allow the gastrointestinal absorption of psychedelic molecules like DMT. Overall, each study successfully used ^1^H NMR spectroscopy to determine the chemical composition of the plants in question, however it is evident that none of these studies used ^1^H NMR spectroscopy to retrieve metabonomic data.

Amariz et al. (87) published a similar paper to study the molecules present in a plant material. The research specifically investigated the *Mimosa tenuiflora*, also referred to as “black jurema.” On this occasion only ^1^H NMR (1D and 2D) spectroscopy and GC-MS were used for analysis. DMT was identified and quantified but interestingly this study compared both analytical techniques to each other and claimed NMR was the best option to quantify the absolute mass of the plant extract. The preference for ^1^H NMR spectroscopy stemmed from the fast analytical processing of this tool and the fact the analytes did not have to be isolated before analysis unlike the preparation for GC-MS. This evaluation of the analytical techniques provides useful information for future studies involved in natural products chemistry. Even though ^1^H NMR spectroscopy successfully identified the molecular composition of the *Mimosa tenuiflora* plant, it was not used for to analyse molecules present in any biofluids.

Pereira et al. (93) studied the *Erythoxylum pungens* plant and were able to identify 12 different molecules, one of them being DMT found in the root of the plant. The molecules were isolated and characterised using ^1^H NMR (1D and 2D) spectroscopy and ^13^C NMR spectroscopy and MS. Once that was achieved, this research group evaluated the cytotoxic activity of the molecules using structure-based and ligand-based virtual screening. They found DMT impaired at 50% the cell viability for HeLa, SiHa, PC3, and 786-0 cell lines; these are human cell lines that are established from cancerous tissue. Despite ^1^H NMR spectroscopy was not used for metabonomics, this is an important publication because it was the first time any psychedelic molecule has been reported in the *Erythoxylum pungens* plant. Therefore, this represents the discovery of a new source of DMT. Additionally, this was the first-time that complete NMR data was collected for this plant.

Additionally, Soares et al. (96) investigated the molecules present in the leaves of *Psychotria vivirdis*. Many different molecules were identified from extracts taken but the only molecule that was relevant for this review was DMT. Structural elucidation was conducted using the standard ^1^H NMR, ^13^C NMR and IR, but they also chose to include GC-MS and LC-MS. For the evaluation of acetylcholinesterase inhibition and cytotoxic activity, this study found that DMT had cholinesterase inhibiting activity and also demonstrated a marked activity against tumour cell strains B16F10 and 4T1. The inclusion of experiments that analysed the biological properties of DMT was useful in producing a better understanding of how the molecule interacts with the rest of the body and not just the neurological effects. However, the use of ^1^H NMR in this study was not to acquire metabonomic data.

It was the research conducted by Tsochatzis et al. (89) in 2020 that identified 1B-LSD using ^1^H NMR spectroscopy. The drug samples were obtained from seized blotter paper at Swedish airport customs, this being the first time this substance was found circulating the street drug market. The seized substances were completely unknown prior to this experiment and were present in small amount, between 30 and 100 ug. The identification and structural elucidation were achieved by using ^1^H NMR and ^13^C NMR (both 1D and 2D), GC-MS and LC-MS. Despite only a small amount of the substance being obtained for analysis, NMR was still capable of identifying 1B-LSD; the analytical profile that was produced in this paper consistent with the work conducted by Brandt et al. (92). It is apparent that ^1^H NMR spectroscopy was capable of replicating the data for the structural identification of 1B-LSD and thus, potentially other NPSs. However, there was no metabonomics approach applied to this particular study, and as such ^1^H NMR was not used for this purpose.

Lenz et al. (90) used ^1^H NMR to analytically characterise the two enzymes a phosphatase (PsiP) and a laccatase (PsiL) present in *Psilocybe cubensis* mushrooms. When this mushroom is injured, it will typically turn blue in colour due to the injury. The mechanism that causes the colour change is poorly understood and it was investigated in this paper. They found that a two-step enzyme cascade occurred in the mushroom to prepare for oxidative oligomerisation of psilocybin which leads to the blue colour. The phosphatase PsiP removes the 4-phosphoryloxy group from psilocybin to produce psilocin, while PsiL oxidises the 4-hydroxy group. The PsiL reaction was monitored *in situ* by ^1^H NMR and ^13^C NMR spectroscopy. This study managed to associate two enzymes with the blue colour change apparent in psilocybe mushrooms, demonstrating that ^1^H NMR can be utilised in molecular chemistry experiments on psychedelic natural products. Despite there not being a metabonomics application for ^1^H NMR in this study, the data produced from these enzymes was useful for future ^1^H NMR studies investigating psilocybin.

Another paper investigating psilocybe mushrooms with ^1^H NMR was conducted by Blei et al. (91) in 2020. They analysed different types of psilocybe species (*P. cubensis, P. cyanescens, P. semilanceata, P.mexicana)* and found a range of molecules in the mushrooms by using LC-MS and stable isotope labelling. They identified harmane, harmine and also a range of β-carbolines as their natural products; β-carbolines are known to be MAOIs. For final confirmation of their results, they purified the two major compounds from *P. cubensis* and did another analysis using ^1^H NMR and ^13^C NMR spectroscopy (1D and 2D); the spectra confirmed the presence of β-carbolines. Their findings clarified the molecules present in the psilocybe mushroom and gave insight into the possible molecular interactions. ^1^H NMR spectroscopy was not used for metabonomics in this paper, but it was used to confirm the structure of molecules in a natural substance.

A metabolite called psilocin glucuronide (PCG) was analytically characterised using ^1^H NMR spectroscopy in a study conducted by Shoda et al. (100). When the psilocybe mushroom is consumed, the psilocin molecule is metabolised by conjugation with glucuronic acid to produce PCG. This metabolite is excreted in the urine of psilocybe mushroom users which has been detected with HPLC previously (107). In this study a synthetic version of PCG was produced by incubating Aroclor 1254 pretreated rat liver microsomes, psilocin and additional cofactors and peptides together for 20 h. The compound structure was then characterised using MS, ^1^H NMR and ^13^C NMR spectroscopy (1D and 2D). The enzymatic synthesis and analysis of PCG in this study could improve identification and quantification of PCG in the urine of psilocybe mushrooms users in future clinical trials. It is evident in this study that ^1^H NMR spectroscopy was not used to metabolically analyse the human urine directly; only HPLC was used for this purpose.

^1^H NMR spectroscopy was used by Costa et al. (103) to identify DMT, 5-HT and the psychedelic molecule called bufotenine (5-hydroxy-N,N-dimetyltryptamine) in the skin secretion of three arboreal amphibian species of the Osteocephalus genus (Osteocephalus taurinus, Osteocephalus oophagus and Osteocephalus langsdorffii) from the Amazon and the Atlantic rain forests. Bufotenin also interacts with the 5-HT_2A_ receptor like DMT. The presence of these psychedelic substances in the skin secretion of these amphibians are speculated to be a chemical defence against predators. The identification was achieved with the use of ^1^H NMR and ^13^C NMR spectroscopy (1D and 2D), HPLC, MS and IR. Through the use of these tools, this study was able to describe bufotenin in the Osteocephalus genus amphibians for the first time. This study did not include the analysis of any human biofluids therefore ^1^H NMR was not used as a metabonomic tool.

A large-scale synthesis of psilocin and psilocybin was conducted by Shirota et al. (104). They produced these psychedelic molecules through only organic chemical reactions and without the chromatographic purification of the psilocybe mushroom. The research found the isolation of the N,O-dibenzyl phosphate intermediate as a zwitterionic derivative was needed to complete the synthesis of psilocybin, and 2D ^1^H NMR spectroscopy was used to identify this intermediate. ^1^H NMR, ^13^C NMR and ^31^P NMR was also used to identify and analyse the structures of the molecules produced at different stages of the experiment, therefore monitoring the molecular changes of the chemical reactions conducted. In this study there was no capacity to include a metabonomics approach as this was a chemical synthesis experiment.

Salamone et al. (105) conducted LSD epimerisation studies using ^1^H NMR spectroscopy. Epimerisation is a conformational change of structure, and LSD is transformed to its isomer form known as iso-LSD, this paper wanted to determine the conditions needed to achieve the equilibrium concentration of this epimerisation. ^1^H NMR was necessary in the integration of the C-9 resonance of LSD and iso-LSD. This study found that regardless of whether the conversion was made from LSD to iso-LSD or iso-LSD to LSD, after undergoing conversion the ratio of the concentrations for LSD/iso-LSD was always 9:1. This study was the first to quantitate LSD epimerisation using ^1^H NMR and it clarified the conditions needed to induce epimerisation in solution. In this study, ^1^H NMR was only used for this purpose once. Due to the nature of this chemistry experiment, the use of ^1^H NMR was strictly for quantification and not metabonomic analysis.

In 1981, ^1^H NMR spectroscopy was used by Migliaccio et al. (106) to compare the conformational properties of the two psychedelic molecules, psilocin and bufotenin. Despite being isomers and both having demonstrated high affinity for the same 5-HT_2A_ receptors (108–112) they have different *in vivo* biological response. In 1968, it was hypothesised by Gessner et al. (113) that bufotenin could not cross the blood brain barrier into the CNS because it had low lipid solubility. Migliaccio et al. decided to use ^1^H NMR spectroscopy to investigate the presence of intramolecular hydrogen bonding in both of these psychedelic molecules, to identify potential structural conformational differences that may be leading to different biological responses. ^1^H NMR spectroscopy was the only spectroscopic tool used but it was unable to produce conclusive results and the difference in biological response was attributed to different pK_a_ values. ^1^H NMR spectroscopy was not used in this study to gather any metabonomic data.

## Discussion

The first aim of this review was to explore the use ^1^H NMR spectroscopy in the field of psychedelic research and determine whether ^1^H NMR spectroscopy has ever been used to investigate the human metabolic response to psychedelic substances with biofluid analysis. The second aim was to assess the potential benefits and limitations associated with ^1^H NMR spectroscopy in this research field. Finally, the third aim of this review was to suggest applications of ^1^H NMR that allow this tool to be used to its maximum potential in future studies and suggest novel approaches that may improve the analytical data retrieved from psychedelic research.

Regarding the first aim, ^1^H NMR spectroscopy has mainly been used for the structural elucidation of psychedelic molecules. The collection of structural and analytical data of psychedelics has played a crucial role for drug discovery research in natural products chemistry. A metabonomics approach has not been used to analyse biofluids and study the effect psychedelic substances has had on the human metabolome with ^1^H NMR. Interestingly, a significant number of papers that were included in this review were published in 2020. This could be an indicator that ^1^H NMR spectroscopy is increasing in popularity as an analytical tool for psychedelic research. However, it is most likely that this is result of psychedelic research having an increase in publication and funding in general, which would lead to an increase in studies with ^1^H NMR but also other techniques like LC-MS and GC-MS. Although, it could also potentially be due to a shift in focus within psychedelic research, so far publications have mainly been focused on the neurological effects of psychedelics with the use of functional magnetic resonance imaging (fMRI) (114–117), electroencephalography (118–120) and magnetoencephalography (67, 121–125). The outcome of this review may suggest the preparation of a new approach in psychedelic research that will increase the use of metabonomics tools to analyse peripheral data from the rest of the body, not just research focused on CNS-related data. That being said, this is only speculative as per the lack of current metabonomics studies in the current literature literature related to psychedelics. It should be highlighted that it is a real limitation to have such a diverse tool such as ^1^H NMR spectroscopy limited to natural products research only.

This review has been able to separate the application of ^1^H NMR in psychedelics to the following four main purposes. ^1^H NMR can be used to: (1) produce an analytical profile for novel LSD derivatives (found in 8 papers); (2) identify psychedelic molecules in natural materials (found in 7 papers); (3) investigate metabolites related to the psilocybe mushroom (found in 3 papers) and (4) analytically characterise psychedelic molecules in their salt form (found in 2 papers). Each of these points will be explored in further detail in this part of the discussion before proceeding to the second aim.

### ^1^H NMR Can Produce an Analytical Profile for Novel LSD Derivatives

LSD derivatives and phenylethylamines contribute to the growing collection of NPSs that are being synthesised and sold in the UK (126). The high number of existing LSD derivatives (64) can often mean that many of these molecules never undergo extensive research; there is typically a lack of analytical data on NPSs and understanding of their mechanisms. This therefore presents a unique application of ^1^H NMR in psychedelic research. ^1^H NMR investigates these LSD derivatives as research chemicals to determine analytical characterisation, biological characterisation and drug safety. In this review, the majority of research into LSD-derived NPSs has been led by Brandt and colleagues (85, 88, 92, 94, 95, 97, 98). Combining all their studies, ^1^H NMR was used to structurally elucidate and characterise nine LSD analogues. These papers successfully created analytical profiles for each drug, and these can serve as a source of reference for any future research groups that desire to investigate them. A real strength of the work conducted by Brandt and colleagues is that they used a wide variety of analytical tools, and it was not just limited to ^1^H NMR or MS. This provides a more holistic profile of the molecules and provides more analytical data than if it were just one spectroscopic method used.

Another interesting aspect of the Brandt *et al*. papers (85, 88, 92, 94, 95, 97, 98) included in this review was that the NPSs were administered to genetically modified C57BL/6 J mice to investigate their biological response. One limitation of animal model studies in psychedelic research is that the psychological effects of psychedelic molecules are hard to determine when the subject cannot express their psychological experience to the researchers. Therefore, the Brandt and colleagues relied on the behavioural response of the mice through HTR that indicates the activation of the 5-HT_2A_ receptors is causing a hallucinogenic effect. It is known that LSD typically causes increased HTR in mice (127) and these papers confirmed that most LSD analogues mimicked this result when administered *in vivo* in mice. Increased HTR response caused by NPS suggest that it has an LSD-like behavioural effect, which is in accordance with these analogues binding to the 5-HT_2A_ receptors.

A limitation of Brandt *et al*. studies (85, 88, 92, 94, 95, 97, 98) are the lack of research into the metabolic breakdown these substances undergo after administration into the mice. An example of such a metabolic analysis can be found in the research conducted by Wagmann et al. (128) where nine LSD derivatives were studied; rats were administered a derivative and their urine was collected. The rat urine analysis was conducted using LC-MS and GC-MS. Unfortunately, no LSD derivatives or metabolites were detected in this particular study, but it indicates that there are potential improvements that could be made to the biofluid analysis methodology in animal studies. Perhaps the Brandt *et al*. studies could have incorporated the collection of the blood plasma of the mice and the analysis of the biofluid through spectroscopic techniques like ^1^H NMR. This data could have then been statically analysed to determine whether increased concentration of the psychedelic molecules in the plasma correlates with increased HTR. If a correlation is observed in the data, this could be proof of concept for the translation of animal studies into human clinical trials and provide an important source of data. NPS research would vastly advance if it could investigate the effect LSD derivatives on humans in clinical trials. However, the ethical approval to use novel substances in clinical trials is an extremely meticulous and difficult process to attain and has to go through years of safety checks in animal trials before transitioning to human trials (129, 130). This would therefore make the transition of LSD derivatives studies in humans more challenging.

Another potential avenue of exploring psychedelic NPSs could be the application of ^1^H NMR spectroscopy to investigate phenethylamines. This review was unable to find any publications that had attempted this application, and so it might be a worthwhile endeavour since there is generally less analytical data about novel phenethylamines like the 2-C substances (131). Overall, the analytical profiles retrieved from NPSs would be essential data if NPSs were ever to proceed to clinical trials. It is necessary in the future to explore the physiological and psychological properties of LSD derivatives and phenethylamines, as they could be alternatives to the psychedelic substances that are currently being investigated for disease treatment.

### ^1^H NMR Can Identify Psychedelic Molecules in Natural Materials

A number of classical examples of natural products chemistry can be observed in the publications included in this review. ^1^H NMR has been used to identify the molecules present in plants by various research groups, resulting in the detection of psychedelics substances like DMT, 5-MeO-DMT and psilocybin. It is apparent from this review that different parts of the plants can have different molecular compositions. Sometimes the psychedelic molecules were only present in the leaf extract, in other cases they were present in the bark. There is no standardised methodology for conducting this type of research, which means that it can be hard to compare the results between different plant species, despite the studies being similar in nature. The findings are still interesting however, it demonstrates that there are multiple sources of psychedelic molecules (especially DMT) from natural products.

Moreover, these papers also identify other non-psychedelic molecules that are also present in these plants. Follow-up research could be undertaken to see the relevance of these non-psychedelic molecules and whether they interact with the psychedelic molecules thus modulating the psychoactive experience; this is known in the field of psychedelic research as the “entourage effect” (132). One psychedelic molecule that has been speculated to have entourage effect is 5-MeO-DMT (133–135). In a paper conducted by Uthaug et al. (136), the effects of the synthetic version of 5-MeO-DMT on mental health was investigated. Findings suggested that 5-MeO-DMT had an impact on several inflammatory biomarkers, whiles also improving affect and non-judgement (*i.e*., a mindfulness related capacity) in volunteers. As part of the discussion, these findings were compared to data from a previous paper the same research group published 1 year prior on the effects of toad secretion containing 5-MeO-DMT on mental health (137). The comparison showed that the consumptions of 5-MeO-DMT (in either form) evoked a similar trend in changes in mental health related parameters, but that those volunteers consuming toad secretion containing 5-MeO-DMT experienced a 20–30% increase in the magnitude for subjective effects like “ego dissolution” and “altered state of consciousness” experienced than what was reported by volunteers who used synthetic 5-MeO-DMT. It is not clear why this was the case, but it is known that toad secretion contains not only 5-MeO-DMT, but also other molecules—though in very low quantities (137). Thus, a potential explanation as to why volunteers that used toad secretion rated the psychedelic experience higher than those who used synthetic 5-MeO-DMT could be because other molecules in the toad secretion are interacting with 5-MeO-DMT to modulate its biological activity like entourage effect predicts (133, 134). However, since the quantity of the other molecules are so low, a more plausible explanation is that other variables may have co-influenced the strength of the experience such as for example, drug concentration in blood, drug metabolism, dose, inhalation technique, facilitation, openness to the experience and/or dysregulation in the nervous system. Nevertheless, this would need to be studied extensively to better understand the plausible existence of such an interaction. The application of metabonomic analysis of biofluids with the use of ^1^H NMR spectroscopy could be beneficial to explore whether the entourage effect exists *in vivo*.

Interestingly, one paper in this review that used ^1^H NMR to analyse the molecules present in the skin secretion of three species of arboreal amphibians. These species were *Osteocephalus taurinus, Osteocephalus oophagus*, and *Osteocephalus langsdorffii* (103). This is another example of natural products chemistry, as the natural products definition not only encompasses plants but any material or substance that derives from an organism. Amphibian skin secretion was analysed with ^1^H NMR spectroscopy and it revealed the presence of DMT, bufotenine (5-hydroxy-dimethyltryptamine; 5-HO-DMT) and 5-HT; all molecules that belong to the tryptamine class. Overall, this paper was useful in broadening the knowledge of potential sources of naturally abundant psychedelic molecules. Moreover, the paper also improves the molecular understanding of toad secretion which could be potential complementary reference for studies on 5-MeO-DMT derived from toad secretion.

### ^1^H NMR Can Investigate Metabolites Related to Psilocybe Mushrooms

There are over 200 types of taxonomically classified psilocybe mushrooms (138), and there were three papers in this review that utilised ^1^H NMR spectroscopy to investigate the molecules associated with the psilocybe mushroom in greater detail. Blei et al. (91) managed to identify in the psilocybe mushroom the molecules harmane, harmine and a variety of β-carbolines. In the Lenz et al. (90) study, two enzymes were found in the psilocybe mushroom that were responsible for converting psilocybin into psilocin when the mushroom experienced traumatic injury. Alternatively, Shoda et al. (100) produced an analytical profile for the molecule psilocin glucuronide (PCG), a metabolite that is found in the urine of psilocybe mushroom users. ^1^H NMR was not used directly on the urine; it was only used on the synthesised molecule of PCG. All three studies were able to improve the molecular knowledge of the psilocybe mushroom through the use of ^1^H NMR spectroscopy.

There is a need to investigate the composition of psilocybe mushrooms as the common administration of the psilocin is through the oral consumption of the whole psilocybe mushroom. It has been reported that there is a strong synergistic interaction between the endogenous molecules in the psilocybe mushroom (139), which might also signify potential entourage effect. This is mirrored in the marijuana plant, whereby the cannabidiol (CBD) molecule interacts with the tetrahydrocannabidiol (THC) molecule to modulate the psychological experience and reduce the social withdrawal side effect caused by THC (140, 141). A psychedelic example of a similar molecular interaction can be observed with the ayahuasca beverage that requires β-carbolines as a MAOI to allow DMT to be absorbed in the gut and exert an effect on the CNS (142, 143). Interestingly, β-carbolines were also found in psilocybe mushrooms (91) and so further investigation could be warranted. These examples highlight the importance of using ^1^H NMR spectroscopy for the complete detection of molecules in the psilocybe mushroom in psychedelic research and this approach could be used to clarify the nature of the molecular interactions.

### ^1^H NMR Can Analytically Characterise Psychedelic Molecules in Their Salt Form

There were two papers in this review that aimed to create salt forms of psychedelic substances. Cozzi and Daley (86) made DMT hemifumurate salt and Sherwood et al. (84) made 5-MeO-DMT succinate salt; these are both molecules that have been used significantly less in clinical trials compared to psilocybin. Previous research investigating consumption of DMT and 5-MeO-DMT in a naturalistic setting noted that the administration for these substances have primarily been through oral administration of ayahuasca for DMT (144) and the inhalation of vapour for 5-MeO-DMT (136, 137). If clinical trials were to be conducted with these psychedelics, the existence of the salt forms is useful because it means there are more options regarding the route of administration. For example, IM or IV injections may be advantageous depending on the trial design. ^1^H NMR spectroscopy has played an important role in determining the drug purity of these salt forms. This is a relevant application of the spectroscopic tool in psychedelic research and could be used for other psychedelic molecule that have a natural substance source and would benefit from being administered through injections. Another benefit of having these papers is that the spectra and analytical data produced by ^1^H NMR can be catalogued for future use and may provide essential information if ^1^H NMR spectroscopy would be used to analyse the biofluids collected from clinical trials experimenting with these salt forms.

### Comparison Between ^1^H NMR and MS for Metabonomics

The second aim of this review was to assess the potential benefits and limitations associated with ^1^H NMR spectroscopy within the field of psychedelic research. From this review it is clear that ^1^H NMR is not currently the preferred technique for metabolically analysing biofluids in psychedelic research. These database searches we carried out as part of this review were not able to find any publications of this nature. The application of MS and chromatography techniques are primarily used to analyse biofluids for concentrations of psychoactive substances (145–148). However, this should not infer that NMR has no place in this research field. MS and NMR are two of the most popular techniques for metabolic studies, both of which bring their own advantages and disadvantages, as seen in Table 2 (149–151). The choice of techniques is largely dependent on the research aim and the nature of the samples, as well as access to instruments that the laboratory may or may not have. MS is more widely used due to its extreme sensitivity to detect molecules (149), this means that from a small sample of biofluid it can analyse a vast number of metabolites. Sample volumes can be as low as 10 ul serum/plasma and this is advantageous in situations where only a small volume of biofluid was able to be collected in the experiment or if one larger sample has to be separated into smaller volumes. Additionally, MS can quantify metabolites at nanomolar and picomolar concentrations (149), which is more sensitive than NMR spectroscopy.

**Table 2:** Comparison between NMR and MS for technical advantages and disadvantages.

	**NMR**	**MS**
Number of metabolites detected	30–150	300–500+ number will vary depending on chromatography technique used
Ability to target specific molecules	Less capable to target	More capable to target
Sensitivity	Less sensitive	More sensitive
Reproducibility	Highly reproducible	Moderately reproducible
Preparation of sample	Sample needs minimal preparation	Sample needs complex preparation
Batch effects	No batch effect on results	Batch effect on results— produces analysis problems
Time required for analysis	Fast process— only one measurement is required to analyse an entire sample	Longer process— time will vary depending on the chromatography technique used
Instrument cost	More expensive to purchase	Less expensive to purchase
Sample cost	Less expensive per sample	More expensive per sample

The advantages of MS are very compelling for research, but the approach has some limitations, and NMR can be utilised in situations where MS approaches fall short. The most apparent downside to MS is that it suffers from batch effects (i.e., the variability to detect metabolites over long periods of time) (150). Advantageously, NMR does not suffer from batch effects to any great extent, and it therefore makes it a more appropriate technique when working with a lot of samples. It should also be considered that MS samples have to come into direct contact with the instrument leading to changes in the measured analyte response over time (150), the unavoidable contact occurs when MS is coupled with another chromatography technique which is the standard procedure. Additionally, MS requires the metabolite to be ionisable if it is to be detected and the technique is destructive because after the samples are analysed they can never be used again. Fortunately, NMR does not have these limitations (152).

There are examples of NMR-based metabonomics used to analyse human biofluids in response to drugs. In one example, ^1^H NMR spectroscopy was used to examine biofluids taken from participants that had been poisoned with xenobiotics such as salicylate, tetrahydrofuran, paraquat, and valproic acid (153). Xenobiotics are exogenous molecules to the human body and are metabolically broken down, and this study was successful in detecting these metabolic consequences in urine by ^1^H NMR. In terms of psychoactive substances, there have been a few notable animal studies that have monitored the effect ketamine has on metabolite levels in biofluids such as urine and blood plasma (154, 155). Indeed, the study by Guo et al. was even successful in comparing the metabolic data retrieved from ketamine-treated mice to gender-based behavioural differences (155). These animal studies establish an experimental design that may be potentially translatable to human studies. However, it is also important to emphasise that ^1^H NMR spectroscopy has been used for the successful metabolic analysis of human urine following the ingestion of 3,4-methyl?enedioxy?methamphetamine (MDMA) (156) and amphetamines (157). Both are psychoactive substances that have been used recreationally by humans and though they are not of the same drug class as tryptamine or phenethylamine psychedelics, they demonstrate a promising capacity for ^1^H NMR spectroscopy to detect psychedelics in human biofluids.

### Future Directions for ^1^H NMR in Psychedelic Research

The comparison between NMR and MS raises an interesting point of contention, namely, why is it worth pursing NMR when MS and other spectroscopic techniques can provide more sensitivity for the detection of many metabolites? The third aim of this review was to suggest applications of ^1^H NMR that allow this tool to be used to its maximum potential in future studies and suggest novel approaches that may improve the analytical data retrieved from psychedelic research. The real incentive to use NMR compared to other metabolomic techniques comes from the potential novel techniques that have branched off from this tool, the main two being real-time NMR and *in vivo* ^1^H NMR (also referred to as *in vivo* magnetic resonance spectroscopy [MRS]). Additionally, the psychedelic research field could also use ^1^H NMR spectroscopy to investigate the effect the gut microbiota may have on the psychedelic experience.

Real-time NMR spectroscopy is a novel approach for NMR-based metabonomics. Typically, NMR spectroscopy would only be used once as a “snapshot” to provide metabolic data from a biofluid at a particular timepoint. Real-time NMR is an innovative approach because it has the capacity to collect metabolic data over several kinetic data points of up to 48 h (158). This could be beneficial to psychedelic research because each psychedelic substance has a specific duration that they evoke psychological, physiological, and biological effect (53). Thus, it could be interesting to monitor metabolite concentration changes over time. The duration varies depending on the psychedelic and the route of administration. For example, when ayahuasca (with 0.6–0.86 mg/kg of DMT) is orally administered it can have an effect on the individual for 4 h but when <100 mg of DMT is smoked it can have an effect for ~30–45 min (159, 160). During the course of the drug effect, real-time ^1^H NMR could be implemented by collecting samples every 7 min, this is a previously used interval (158, 161). Theoretically, this should produce enough kinetic data points to provide detailed information about metabolite concentration fluctuations over time. This may lead to more accurate correlation between psychological experience and metabolic data collected. Metabolomic studies that use spectroscopic tools like MS or chromatographic tools do not have the capacity to do real-time analysis to the same efficiency as NMR spectroscopy, this is because NMR has minimal sample preparation and relatively rapid sample acquisition (152, 162), which means a study with a multitude of samples can be readily analysed. This unique potential that NMR has for real-time analysis provides strong incentive to successfully integrate NMR spectroscopy into psychedelic research.

The obvious major set-back with real-time NMR spectroscopy is the numerous biofluid collections required to retrieve data at different kinetic time points. Different methods of biofluid collection exist and each vary in invasiveness. It is important to select the correct collection method to minimise participant stress and/or discomfort. One viable non-invasive option is the collection of urine, which has advantages due to urine being sterile and easy to obtain in large volumes (163). Compared to other biofluids it also has far more metabolites with greater chemical diversity (152). The most notable problem with urine sampling is that it is difficult to control when participants need to urinate, and this would mean that kinetic points for each individual may distinctly vary. Of course, it is possible to approximate time the urine samples were collected before and after substance administration, but this would rule out the possibility of real-time NMR that requires multiple and time-specific samples. Another additional limitation is that it can take time for metabolites to appear in urine (164), the data retrieved may not be ideal for studies investigating correlations with subjective reports (questionnaires). In summary, urine may not be the most optimal biofluid to collect. Another alternative biofluid to consider for real-time NMR spectroscopy is saliva (165), as it is a biofluid that can be collected at specified time points repeatedly. In fact, saliva ^1^H NMR studies is an underutilised approach even outside psychedelic research despite demonstrating promising metabolite quantification (166). Saliva samples are usually collected in tubes (165), however, it is possible that the effect of psychedelic substances may mean that participants are unable to spit. Saliva can also be collected using swabs (165), but there is the possibility that this is still too disruptive for the participants, so perhaps alternative biofluid options could be explored. Blood plasma is another biofluid that can be used for NMR analysis (167). In terms of discomfort, blood collected through a cannula causes little discomfort (168), because it only requires one insertion at the start of the experiment and samples are easily accessible. This could make a potentially good candidate for metabonomics studies.

An alternative avenue for NMR related research is *in vivo* ^1^H MRS, this tool does not analyse biofluids but instead measures the biochemical changes directly in the brain. ^1^H MRS can be conducted by placing a participant in a magnetic resonance imaging (MRI) scanner that has been set up with additional software and hardware for the acquisition of MRS data (169). In MRI, water molecules are detected to produce a spatial map of the brain, but for MRS the focus is on detecting metabolite signals in specific brain regions (typical voxel size: 4–8 cm^3^) (170); these metabolites can be neurotransmitters like glutamate (171–173) or γ-aminobutyric acid (GABA) (174–176). The psychopharmacological approach to ^1^H MRS involve studies that monitor neurotransmitter changes in response to anti-depressant medication (177–179), and similarly, this approach can be used to study psychedelics. In 2017, an open label uncontrolled ^1^H MRS study was conducted to measure metabolite change in the brain 24 h after (post-acute) administration of ayahuasca (containing 0.64 mg/kg of DMT) in healthy volunteers (180). MRS results showed the posterior cingulate cortex (PCC) had a reduction of glutamate, glutamine, creatine, n-acetylaspartate, and n-acetylaspartylglutamate. Volunteers completed a mindfulness questionnaire, and the reduction of both glutamate and glutamine (glx) was correlated with an increase in the non-judging subscale score. It was speculated that the post-acute decrease of glx in the PCC could be a result of excitatory activity increasing during the acute phase, unfortunately the MRS was not conducted during the acute phase to confirm this but the hypothesis is in agreement with past rat studies that found that psychedelics have an excitatory effect on neurons that project to the neocortex (53), which would include the PCC region. Overall, this MRS study is important as it has endeavoured to connect neurochemical changes with the psychological experience of ayahuasca. The magnetic field strength needed to obtain spectra in this study was 3 Tesla (T). This is limitation because 3T studies are out of date with the popularisation of MRS studies that use better superconducting magnets. ^1^H MRS studies can now safely use higher magnetic field strengths of 7T (181) and beyond (182, 183). These studies are called ultra-high-field (UHF) studies and have a better signal-to-noise ratio (184), which improves the reliability of the spectra compared to 3T. UHF studies can be associated with side effects like nausea, dizziness and a metallic taste (181) which is often caused if the participants has moved in or out of the MR machine too quickly, but these effects do not occur if the individual is static in the machine and does not appear to be a major concern between 7T and 9.4T (181).

Therefore, a notable advancement is the use of ^1^H MRS at 7T to investigate the neurotransmission changes in response to psychedelics. In 2020, Mason et al. conducted a double-blind placebo-controlled study to investigate the acute effect psilocybin had on glutamate neurotransmission in the medial prefrontal cortex (mPFC) and hippocampus (185). In addition to ^1^H MRS (7T), fMRI was used for neuroimaging and the psychological subjective state of participants was assessed using questionnaires for ego dissolution and altered state of consciousness. Psilocybin was given to healthy volunteers in a low to moderate dose (0.171 mg/kg). Results found that brain region-dependent glutamate changes were a strong predictor to changes in ego dissolution, for example higher glutamate levels in the mPFC was associated with negatively experienced ego dissolution, whereas lower glutamate levels in the hippocampus was associated with positively experienced ego dissolution. The outcome of the mPFC is congruent with a previous ^1^H MRS study that has demonstrated a link between higher glutamate levels in the frontal cortex and increased anxiety (186). Additionally, another study has previously hypothesised that the hippocampus is involved in ego dissolution as a result of psychedelics causing temporary loss of access to semantic autobiographical information (187), therefore allowing the breakdown of personal identity which coincides with the results produced by Mason et al. This is a good example of how ^1^H MRS can be used to provide more complex correlations that indicate a neurochemical basis to the psychological effects experienced by healthy participants in psychedelic research.

Further studies could build upon this to understand the effect psychedelics might have for disease treatment. In 2019, Barrett et al. conducted a study using ^1^H MRS on major depressive disorder (MDD) patients (188), who underwent two sessions of psilocybin at higher doses and were monitored for changes in the anterior cingulate cortex (ACC) and hippocampus weeks after the administration. This study was able to conclude that glutamate was decreased in the ACC and increased in the hippocampus. This was interesting because the MDD patients also had improved cognitive flexibility suggesting that there may be a relationship between the changes in glutamate in these brain regions and anti-depressant effects of psilocybin. In the future, similar studies could be conducted with a placebo control or against a serotonin reuptake inhibitor drug that serves as the standard anti-depressant treatment, as this would assess the benefits of psychedelic assisted therapy using ^1^H MRS. Overall, there have been major advances to the application of the *in vivo* MRS technique in neuroscience which is largely due to the more powerful superconducting magnets. However, there are still further improvements that could be made to the methodology of MRS and this is paramount since—to the best of our knowledge—no psychedelics studies so far have used improved methodological techniques like functional ^1^H MRS in combination with 7T. Functional ^1^H MRS involves sequential scans being collected whilst participants are exposed to a stimulus/task-based condition which allows for “dynamic” neurotransmitter release to be measured (189), this provides more reliable findings than static basal level measurements that ^1^H MRS studies are conventionally used. This would mean scans could be taken when a pain stimulus is non-invasively inflicted by either cold-pressure or acute heat (189) or an alternatively during a flashing checkerboard stimulus where a flashing light is shone into the participants eye to stimulate glutamate release (189). Advancements in both the magnetic field strength and methodology of ^1^H MRS could see this technique used with more frequency in the future of psychedelic research and could be a beneficial addition to traditional imaging techniques like fMRI.

A final suggestion for future research is to investigate the influence of the gut microbiota on the effect of psychedelic molecules. ^1^H NMR spectroscopy is heavily used in gut microbiome studies (190–194). One way to investigate this interaction would be to compare the different routes of administrations for a psychedelic, for example oral administration of ayahuasca that contains DMT may have a different biological effect compared to intramuscularly injected DMT alone; the former does not only include DMT but also MAOIs and secondly it interacts with the digestive system while the latter does not. Even the speed at which the psychedelic substances are orally administered is worth considering for gut microbiota studies. For example, in the book PiHKAL (195) it is mentioned that when the peyote cactus is orally administered quickly it will cause substantial nausea. The peyote cactus contains the psychedelic mescaline and when synthetic mescaline is also consumed quickly people have reported the same nausea. Shulgin notes that when the peyote cactus or synthetic mescaline is slowly consumed over a 30 min period the nausea is completely absent, thus negating this negative side effect. Clearly the molecule is engaging differently with the digestive tract when the speed of the oral administration is elongated, and this hints to a potential meaningful interaction between psychedelic substances and the ENS. Gut microbiota studies could even be used to better understand the implications of traditional psychedelic practises. Individuals will typically undergo a diet that excludes salt, sugar, spices, oil and meat, abstain from alcohol and even sex prior to the psychedelic ceremonies (196). This “purification” practise is used mainly for ayahuasca ceremonies because it is believed to maximise the physiological effect (196). It is possible that this cleansing changes the gut microbiota and allows for better absorption or has consequential effects on the gut-brain axis (197–200). ^1^H NMR could be used to investigate this further and discern the pharmacodynamics of psychedelic substances in the ENS, which could lead to a better understanding of the molecules effect on the CNS and thus, the development of better novel treatments for psychiatric conditions.

## Conclusion

This review highlighted that the main use of ^1^H NMR was for the structuralelucidation and analytical characterisation as required in natural products chemistry and it was used as a complimentary method to MS and chromatographic tools. There were no papers that used ^1^H NMR in the metabolic profiling of biofluids (i.e., blood, urine, and saliva) and as this technique has the capacity to study the biological effects that accompany the psychedelic experience it could be beneficial in future clinical and naturalistic observational research. The absence of batch effect and relatively easy sample preparation in NMR techniques improves its efficacy on metabonomic studies; however, for biofluid analysis it would be recommended the use of multiple spectroscopic tools that complement the data and ensure that individual technique limitations are minimised.

Furthermore, we emphasised the importance of the potential use of real-time NMR in biofluids and *in vivo* ^1^H MRS of specific brain regions (preferentially at magnetic field strength of greater than or equal to 7T) to monitor changes in metabolite concentrations in response to psychedelic molecules before, during and after administration. Additionally, we also suggest that the extensive use of ^1^H NMR in the study of the gut microbiota should now focus on the biological interaction of the gut-microbiome with psychedelic substances.

In conclusion, we believe that ^1^H NMR is an assessment approach worthy of consideration for researchers and the field so to expand the knowledge of the biological effects of psychedelics. This could allow for a better understanding of the subjective psychedelic experience reported by individuals, which could in turn allow for improved treatment options that minimise the adverse side effects and maximise the therapeutic effect for mental health disorders such as depression and autoimmune disorders. The biochemical data retrieved could be a critical addition to psychedelic research.

## Author Contributions

SV-M conducted the literature review and wrote the manuscript. JG and MU provided feedback and edits for this manuscript. All authors approved the final manuscript.

## Funding

Work in the JG laboratory is supported by the Medical Research Council (MR/P011705/1 and MR/S010483/1) and the Dementia Research Institute (UKDRI-5002). MU efforts were financially supported by Eric Grotefeld and Ryan Zurrer. Eric Grotefeld and Ryan Zurrer had no role in the decision to publish, or in the preparation of this manuscript.

## Conflict of Interest

JG receives research funding from Astra Zeneca and Unilever. These companies did not have any role in the decision to publish, or the preparation of this manuscript. The remaining authors declare that the research was conducted in the absence of any commercial or financial relationships that could be construed as a potential conflict of interest.

## Publisher's Note

All claims expressed in this article are solely those of the authors and do not necessarily represent those of their affiliated organizations, or those of the publisher, the editors and the reviewers. Any product that may be evaluated in this article, or claim that may be made by its manufacturer, is not guaranteed or endorsed by the publisher.
